# Modelling of Soybean (*Glycine max* (L.) Merr.) Response to Blue Light Intensity in Controlled Environments

**DOI:** 10.3390/plants9121757

**Published:** 2020-12-11

**Authors:** Tina Hitz, Simone Graeff-Hönninger, Sebastian Munz

**Affiliations:** Institute of Crop Science, Cropping Systems and Modelling, University of Hohenheim, 70599 Stuttgart, Germany; tina.hitz@uni-hohenheim.de (T.H.); graeff@uni-hohenheim.de (S.G.-H.)

**Keywords:** photomorphogenesis, blue photon flux density, functional structural plant modelling, indoor farming, LED lighting

## Abstract

Low photosynthetic photon flux density (PPFD) under shade is associated with low blue photon flux density (BPFD), which independent from PPFD can induce shade responses, e.g., elongation growth. In this study, the response of soybean to six levels of BPFD under constant PPFD from LED lighting was investigated with regard to morphology, biomass and photosynthesis to increase the knowledge for optimizing the intensity of BPFD for a speed breeding system. The results showed that low BPFD increased plant height, leaf area and biomass and decreased leaf mass ratio. Photosynthetic rate and internode diameter were not influenced. A functional structural plant model of soybean was calibrated with the experimental data. A response function for internode length to the perceived BPFD by the internodes was derived from simulations and integrated into the model. With the aim to optimize lighting for a speed breeding system, simulations with alternative lighting scenarios indicated that decreasing BPFD during the growth period and using different chamber material with a higher reflectance could reduce energy consumption by 7% compared to the experimental setup, while inducing short soybean plants.

## 1. Introduction

In horticulture and indoor farming, LEDs have several advantages e.g., they save energy, emit less heat and have a long lifetime [1,2]. A spectrum can be designed depending on the response of the specific crop and the production aim. However, to fully exploit the spectral flexibility of LED lighting an increased knowledge of the spectral effects on plant morphology and growth is required [3]. Energy consumption can also be considered during spectral optimization as this can vary between spectra depending on the LED types [4]. A higher energy consumption of red than of blue LEDs has been reported [4,5], but theoretically the energy consumption of blue LEDs is higher than of red LEDs due to the higher energy level per photon of shorter than of longer wavelengths [6].

The advantages of LED lighting can be used in speed breeding, a breeding system developed particularly for growth chambers. The aim of a speed breeding system is to grow many generations per year to shorten the time for developing new cultivars. For instance, in a speed breeding system for several cereals, pea and chickpea six generations can be grown per year [7]. For a more efficient use of space, plants can be grown in a multi-layer system. For these systems, short plants are desirable to increase the number of layers of plants and hereby the possibility to include more genotypes at the same time. Therefore, a spectrum for speed breeding should not delay seed setting (many generations) and induce a shorter plant height to cultivate in more layers (many genotypes). These requirements deviate from other indoor plant productions aiming to increase resource use efficiency considering other properties, such as yield and nutritional value [5]. Recently, a speed breeding protocol for soybean was developed using LED lighting. Red and blue light was found not to influence flowering time and was recommended to induce short compact plants. However, only two ratios of red and blue light (1:1 and 2:1) were studied [8].

The spectral light environment is perceived by the plant photoreceptors, which in a natural environment induce morphological changes such as those that express shade adaptations [9]. Shaded plants experience a reduced red to far-red ratio perceived by phytochrome [10] and a reduced photosynthetic photon flux density (PPFD). The latter is associated with a reduced blue photon flux density (BPFD) perceived by cryptochrome [11]. Typical shade responses of soybean are elongated internodes and petioles, increased specific leaf area (SLA) and decreased biomass and internode diameter [12,13,14]. Under LED lighting, BPFD can be reduced by lowering the ratio of blue light in the spectrum without a simultaneous reduction of PPFD. By reducing BPFD, some morphological shade responses, e.g., increased height, can be triggered also under constant PPFD [15,16]. For soybean, earlier studies found an increased plant height with decreasing BPFD [17,18,19] showing that high BPFD can be applied to induce short soybean plants, but these studies used a broad spectrum and included only one treatment [17] with a blue light ratio over 30%. None of these studies derived a response function to BPFD for soybean height under LED lighting with narrow peaks and none focused on blue light ratios between 15–78%. Earlier studies in other species than soybean also explored relatively low BPFD ratios (<50%) with the aim of avoiding extreme elongation under sole red LED lighting [15] or explored an intermediate BPFD to maximize biomass [20]. The aim in the present study was to reduce plant height to its minimum under a high BPFD.

Beside the influences on plant growth through the perceptions of photoreceptors, the light spectra can also influence the photosynthetic rate. Whereby, carbon assimilation can differ depending on the spectrum even under a constant PPFD. Photosynthetic pigments of plants absorb light mainly within the range of wavelength from 400 to 700 nm. The photosynthetic most effective part is considered to be the light within the red range (600–700 nm) due to a better balance of excitation between photosystem I and II and due to a more effective transfer between the red light absorbing chlorophylls than from the blue light absorbing carotenoids to chlorophyll [21]. Despite this, several studies measuring photosynthesis on plants grown under different light spectra found similar rates of photosynthesis under spectra with different ratios of light within the blue range (400–500 nm) [22,23,24].

The optimization of light spectrum for a specific crop and production system is very time-consuming given the many aspects that have to be considered, e.g., light quality, intensity and day length. Also, the transfer of knowledge between studies and into practice can be impaired by variability in several factors, e.g., plant density, type of light source and dimensions of the climate chambers. In this context, functional structural plant (FSP) modeling can assist as a tool for optimization of crop production and understanding of plant responses to its environment. An FSP model simulates plant growth and development, while considering its architectural appearance, by responding to the experienced environment on the individual organ level [25,26]. Hereby, responses can be related to the actually perceived spectrum of individual organs. The perceived spectral light environment can differ from the environment above the canopy and between phytomers due to self-shading and light reflection, as other studies found focusing on PPFD [27,28,29] or the red to far red ratio [30,31,32,33,34].

Earlier FSP models using artificial light sources for indoor plant production addressed the light regime for greenhouse production [35,36,37], while only one study used an FSP model with LEDs being the only light source [38]. An FSP model within an LED chamber can be a tool to reduce the amount of necessary experiments for spectral optimization and assist in the understanding of the plant response to the indoor environment and in the transfer of knowledge between studies and into practice.

The aim of this study was to find an optimal BPFD inducing short soybean plants under a narrow peaked red and blue LED spectrum, also considering energy consumption. We hypothesize that an optimum BPFD for minimum plant height, not influencing flowering time, could be determined with a combination of experiments and FSP modelling. The objectives were to (i) examine the influence of different levels of BPFD under constant PPFD on soybean biomass, photosynthesis and morphology and (ii) calibrate an FSP model of soybean and integrate a response function to BPFD for internode length and (iii) to find by simulation the minimum BPFD to reduce plant height and energy consumption.

## 2. Results

### 2.1. Experimental Data—Plant Scale

**Figure 1:**
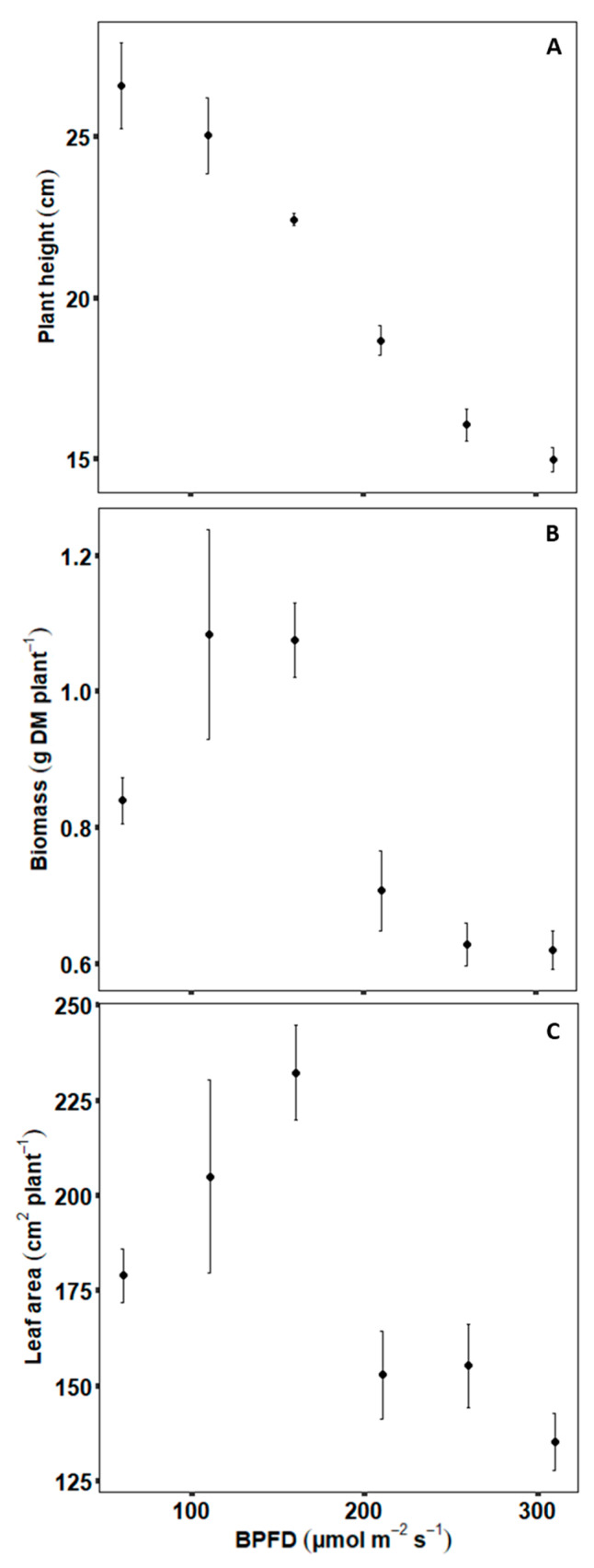
Final plant height (**A**), biomass (**B**) and leaf area (**C**) under different blue photosynthetic flux densities (BPFD). Error bars indicate standard error of the mean (*n* = 8).

Biomass and leaf area per plant showed similar differences among the six light treatments with a BPFD of 60, 110, 160, 210, 260 and 310 µmol m^−2^ s^−1^ (B60–B310). The treatments B110–B160 resulted in the highest values and B210–B310 in the lowest values, whereas plant height consistently decreased with increasing BPFD (Figure 1).

Plant height responded rapidly after beginning of the experiment and the differences between treatments became more pronounced over time with differences between the highest and lowest values of 34%, 46%, and 34% on day 9 and 77% 75% and 72% on day 23 for plant height, biomass, and leaf area, respectively (Supplementary Materials, Figure S1).

### 2.2. Experimental Data—Phytomer Scale

#### 2.2.1. Biomass

At the third phytomer, significant differences between treatments were found for biomass of internodes and leaf laminas. An increase in BPFD decreased the biomass of internodes and leaf laminas with the minimum of 0.022 and 0.078 g under B260 and the maximum of 0.046 and 0.136 g under B160. The same tendency was found for biomass of the second internode and the petiole, with the latter being less expressed with no significant differences between treatments. The leaf mass ratio (LMR) differed significantly between BPFD levels with a minimum value of 0.64 under B160 increasing to 0.69 under B60 and 0.71 under B310. The internode mass ratio of the stalk (IMRS) decreased from 0.72 under B260 to 0.63 under B60 (Table 1).

#### 2.2.2. Leaf Morphology and Physiology

At the third phytomer, the SLA significantly increased from 303 under B110 to 346 cm^2^ g^−1^ under B310. No significant differences were observed for carbon assimilation, but there was a slight reduction with increased BPFD from 28.81 µmol CO_2_ m^−2^ s^−1^ under B110 to the minimum assimilation of 26.53 µmol CO_2_ m^−2^ s^−1^ under B310. SPAD values did not differ significantly between treatments and showed no tendency (Table 2).

#### 2.2.3. Elongation

**Figure 2:**
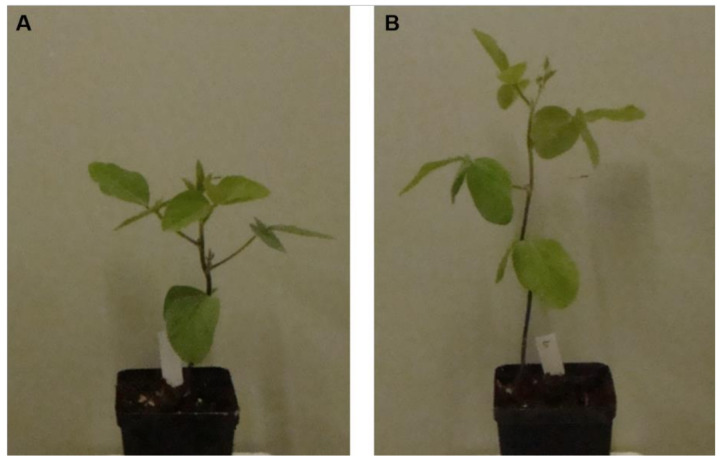
Soybean grown under B310 (**A**) and B60 (**B**).

The internode responded more to a decrease in BPFD at the third than the second phytomer with a length of 2.14 cm under B310 and 3.81 cm under B60, corresponding to a 78% increase (Figure 2; Table 3). Whereas, the second internode increased from 4.33 to 6.86 cm, corresponding to 59%. The response of the petiole was smaller with a length increase from 4.46 cm under B310 to 5.61 cm under B160, corresponding to a 26% increase. The tendency differed from that of the internodes with a maximum length under B160 and an insignificant decrease until B60. Increasing BPFD from B160 also decreased the length of the petiole, but with no significant differences from B210 to B310. Length of the leaf lamina did not respond significantly but had a tendency to decrease from B160 to B310, similarly to the petiole. The internode elongation was not accompanied by a reduced internode diameter, which showed no significant differences with a slight tendency of responding similar to the petiole.

#### 2.2.4. Growth Dynamics

Growth of the individual organs was fitted to the beta-function and parameters for the third phytomer showed significant differences for internode and petiole, but not for the leaf lamina. The absolute differences of the parameters for all three organs were relatively small and did not show any tendency to change with decreased BPFD (Table 4).

#### 2.2.5. Energy Consumption

The highest energy consumption was measured under a high BPFD (Table 5). The consumption increased from 94.4 W under B60 to 107.2 W under B310 corresponding to an increase of 14% under B310.

### 2.3. Modelling

Simulations based on the found parameters of the beta-function resulted in simulated length of internodes, petioles and leaf laminas following the measurements well (exemplified by the treatments B60 and B310 in Supplementary Materials, Figure S2).

#### 2.3.1. Blue Light Response Function of Internodes

**Figure 3:**
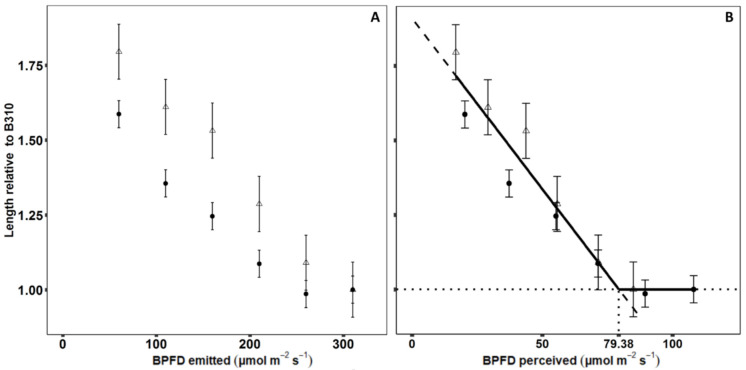
Least square mean of length of second (point) and third (triangle) internode relative to B310 in response to BPFD emitted by the LED modules (**A**) or simulated BPFD perceived by the internode (**B**). Dashed line showing the function fitted to relative internode lengths higher than one and dotted lines showing the interception of the function with 1. Black line showing the final response function to BPFD. Error bars indicate standard error of the LS-mean (*n* = 8).

Based on the simulated *BPFD_per_*, the relative elongation response of the second and third internode were closer to each other compared to using the emitted BPFD (Figure 3). Especially at high BPFD levels, the response of the two internodes was close, implying a common response function to *BPFD_per_*.

Due to the relatively small differences between the parameters *t_e_* and *t_m_* of the beta-function and no clear tendencies in their response to BPFD, only differences in the final length of the internode *(L_max_)* were considered in the response function. Internodes with a relative length to B310 below one were considered to have no elongation response to *BPFD_per_* and a common function for final internode length was fitted to the *BPFD_per_* of the internodes under treatments with a relative length to B310 higher than one (Figure 3B).

The common function for the relative length of the second and third internode was:

(1) $$L_{rel}=-0.01\;*\;BPFD_{per}+1.91,$$

The interception of this function with one was at 79.38 µmol m^−2^ s^−1^ *BPFD_per_*, which was hereby the minimum amount of BPFD that an internode should perceive to express no elongation response to *BPFD_per_*.

This resulted in the BPFD response function:

(2) $$Internode\;length=L_{min}\left(1+(79.38-BPFD_{per}\right)\;*\;0.01),\;79.38-BPFD_{per}>0,$$

where *L_min_* is the final internode length with no elongation response to *BPFD_per_* and *BPFD_per_* is the BPFD perceived by the internode. The black line in Figure 3B shows the response function in the range from the minimum (16.78 µmol m^−2^ s^−1^) to maximum (108.13 µmol m^−2^ s^−1^) perceived light during the simulations.

#### 2.3.2. Evaluation and Light Optimization

**Figure 4:**
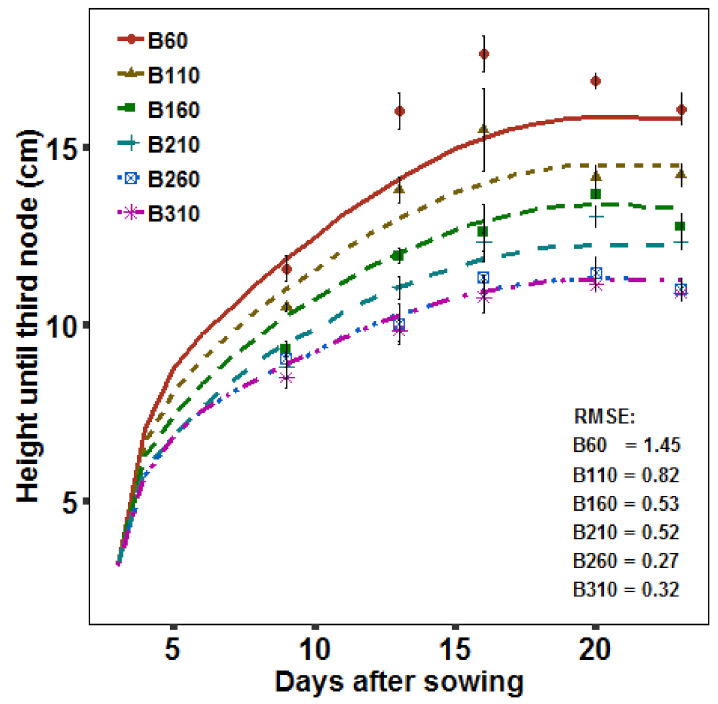
Simulated (line) and measured (points) plant height until the third node and root mean square error (RMSE) between simulations and measurements. Error bars indicate standard error of the mean (day 9–20: *n* = 4, day 23: *n* = 8).

During the simulations based on the found response function, *L_min_* and the growth parameters *t_e_* and *t_m_* were set according to the treatment B310 (baseline scenario). The simulated height until the third internode fitted well with the measurement at the last day, which was also used for parameterization of the model. This shows that the response function was well integrated in the model. Comparing to earlier measurement days which were not used for parameterization of the model, the simulations had a tendency of underestimating the height under low BPFD levels (B60–B110). Importantly for the alternative scenarios the simulated height until third internode fitted well under the higher BPFD levels (B160–B310) (Figure 4).

**Figure 5:**
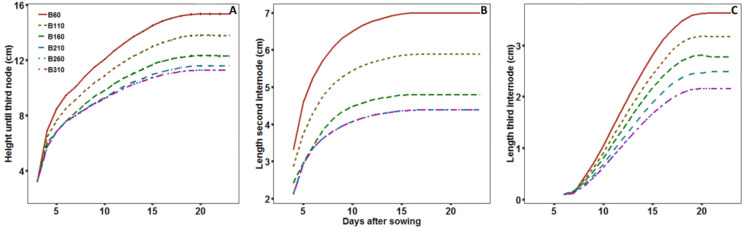
Simulated height until the third node (**A**) and length of second (**B**) and third (**C**) internode with a reflective surface of pots, soil and bottom.

The simulations from the first scenario with a reflective surface of pots, soil and bottom resulted in an increase of perceived BPFD. The total height until the third node decreased under all treatments (Figure 5A) compared with the experimental chamber design (Figure 4). In the experimental design, the minimum height was reached between B260 and B310, while in the alternative scenario it was reached between B210 and B260. The simulated length of the second and third internode (Figure 5B,C) showed that the shorter height was a result of an increase in the perceived BPFD of the second internode, where the minimum length was already reached between B160 and B210 (Figure 5B).

The optimization indicated by the first scenario was applied in the second scenario by optimizing BPFD during the growth period. When the third internode started to develop the treatment changed from B210 to B260 and hereby increased BPFD. The results of the second scenario showed that increasing BPFD on day nine resulted in the minimum length of both internodes and the minimum height of 11.28 cm until the third node. The reduction in the average BPFD emitted by the LED modules resulted in a reduction of energy consumption from 107.2 W in the experimental scenario to 101.7 W in the first and 100.1 W (−7%) in the second scenario (Table 6).

## 3. Discussion

### 3.1. Biomass and Photosynthesis

During the experiments, data on photosynthesis and biomass was collected. This data shows the response of carbon assimilation and translocation to BPFD, which is of minor importance in a speed breeding system, but of interest for improving yield in indoor farming.

No significant influence on carbon assimilation per leaf area was observed with increased BPFD, which is in agreement with earlier studies, although red light is considered to be the most effective for photosynthesis [21,39]. An increased maximum assimilation with increased BPFD in cucumber was associated with an increased leaf thickness [16,22]. Similar, the tendency of decreased assimilation in this study was associated with thinner leaves under high BPFD. In ice plant He et al. [23] found no change in saturated assimilation between BPFD ratios of 10 and 100%. Although an increasing BPFD ratio from 0 to 20% increased photosynthesis in lettuce, it dropped again at 30% [24]. In this study, the lowest BPFD ratio was 15% under the B60 treatment and an effect below this ratio cannot be excluded.

The decrease in biomass at high BPFD found in this study was most probably related to similar differences in leaf area, which decreased light interception and consequently carbon assimilation per plant. Another reason could be an increased root biomass, but earlier studies found no change in the biomass ratio of soybean under BPFD ratios of 10 and 25% [19]. In addition, an influence of BPFD on the assimilation over time could reduce biomass. For instance, the photosynthetic rate of tomato decreased more in the afternoon under monochromatic red and blue light than under a broader spectra [40].

Increased LMR under high BPFD confirming earlier results [18,19] indicated a reduced carbon export from the leaves. In tomato, light spectra also influenced the ratio of carbon export from the leaves, but not in agreement with this study as export increases under monochromatic blue and orange light at intermediate PPFD [40]. This can be caused by different responses comparing monochromatic spectra with broader spectra exploring ratios between wavelengths. A decreased fraction of the carbon translocated from the leaves to the stem (internode and petiole) was located in the internodes (low IMRS) under increased BPFD. These results of biomass proportion between organs showed, that an elongation response to reduced BPFD increased the translocation of carbon from the leaves to the stem, but with a higher priority of internodes than petioles. Extensions of the FSP model could assist in the exploration of carbon assimilation and translocation between organs following a similar approach as Bongers et al. [33] combining response functions to light environment with increased carbon demand of specific organs.

### 3.2. Response to BPFD Under Shade

Low BPFD in Nature is associated with low PPFD and low red to far-red ratio under shade, which trigger morphological responses, e.g., by interactions of the photoreceptors cryptochrome and phytochrome, to increase light interception [41]. The performed experiments represented unnatural spectra that do not occur in nature and hereby show the response of soybean to BPFD without interactions with PPFD and red to far-red ratio.

The elongation response of internode and petiole to low BPFD was in accordance with a shade avoidance response of soybean to low PPFD [12,42] and show that low BPFD can trigger the response also under high PPFD and in the absence of far-red light. The stronger response of internodes than of petioles supports earlier indications of internode elongation being the main shade avoidance response to low PPFD (associated with low BPFD), whereas petiole elongation responded strongly to low red to far-red ratio [12]. The slight decrease in SLA under low BPFD in this study is not in accordance with earlier studies in soybean, which found no response to BPFD in SLA under high PPFD [18,19]. This could be an effect of the lower maximum BPFD ratios applied in earlier studies. Cucumber under low BPFD responded with an increased SLA, which indicates differences between species or an effect of the lower light intensity (100 µmol m^−2^ s^−1^) applied in these studies [16,22]. Decreased SLA and unchanged internode diameter under low BPFD differ from the soybean response to low PPFD resulting in increased SLA [12,18,19] and decreased internode diameter [12,42]. This indicated that SLA and internode diameter are not regulated by the perception of low BPFD associated with low PPFD, but instead supports earlier studies indicating that SLA is regulated e.g., by sugar signaling [43,44,45].

### 3.3. BPFD Response Function

A linear function described well the response to BPFD and was applied for the simulations. Kahlen and Stützel [46] also applied a linear response to PPFD and red to far-red ratio for modeling the response of cucumber to light environment. Other studies found a non-linear response function to BPFD for stem length of soybean [17,18,19]. This can be due to lower BPFD levels in these studies (BPFD levels < 5%) based on which a non-linear function could be fitted [19]. A continuation of the function in the present study below a BPFD ratio of 15% could evolve non-linear, but in the context of speed breeding this low BPFD levels are not important as this would result in tall plants. For the speed breeding system, it was important to determine the point of a saturated response to BPFD to reach short plants and reduce the BPFD to reduce energy consumption. A saturated response to emitted BPFD was reached under treatments between 210 and 310 µmol m^−2^ s^−1^ in the experimental setup. Two earlier studies on soybean found a saturated response already under 30–50 µmol m^−2^ s^−1^ BPFD [17,18], whereas one study also found an effect from higher BPFD (130 µmol m^−2^ s^−1^) [19], indicating interactions with other factors resulting in these discrepancies. One aspect could be the light spectrum, as earlier studies used broader spectra containing green and far-red light [17,18,19] and additionally included UV-A light in the BPFD [19]. Green light can influence cryptochrome antagonistic to blue light and especially under high PPFD [47]. The addition of green light to a red and blue spectrum increased plant height of soybean under a PPFD of 200 µmol m^−2^ s^−1^ but had no influence under 500 µmol m^−2^ s^−1^ [48]. Far-red light can lead to an increase in plant height by reducing the red to far-red ratio perceived by phytochrome as shown for soybean by adding far-red light to a broad light spectrum [12]. In addition, a broader spectrum within the blue range can influence the magnitude of the blue light effect on cryptochrome. For hypocotyl elongation of *Arabidopsis thaliana* (L.) Heynh., the action spectrum of cryptochrome to monochromatic light did not change within the range 390–530 nm, but an increased stability of CRY2 protein was observed under monochromatic light compared to a broader blue spectrum [49]. These differences in the reactions under narrow peaks compared to the reactions during the response to high PPFD, here imitated with high BPFD, indicated that a broader spectrum within the blue range could affect the BPFD level necessary to avoid an elongation response.

In the experiments, the elongation response of the third internode to low BPFD was slightly stronger than at the second internode, and a higher BPFD level was necessary to achieve the minimum length of the third internode. Simulations indicated that this was due to self-shading, which was larger at the third than the second internode. Based on the simulated *BPFD_per_*, a common response function was found for the second and third internode. This emphasizes the importance of knowing the perceived light environment at organ-level, e.g., as in this study by means of simulations with an FSP model, as it enables a better evaluation of the influence from the light microclimate than relating the response directly to the light emitted from the light source [46].

The parameters *t_e_* and *t_m_* of the beta-function were in most cases not significantly different between treatments and no trend was present, which indicated that a common parameter could be used for all levels of BPFD only changing *L_max_* according to the *BPFD_per_*. This was confirmed by the accurate simulations of internode length at all BPFD levels based on *t_e_* and *t_m_* found under B310. Importantly, the small difference in height between B310 and B260 were well simulated, showing that the model was very useful to determine the necessary BPFD to reach the minimum height.

### 3.4. Optimization of Light Spectrum

The decreased biomass with increased BPFD is in accordance with earlier studies in soybean [19] and other species [5,23]. This results in a decreased efficiency of the applied PPFD in indoor farming producing biomass, but in a speed breeding system there are no apparent advantages of a high biomass. Further consideration for a spectral optimization would be whether the minimum necessary BPFD found here could be reduced through other light microclimatic factors. Light intensity would be an important factor to determine possible interactions between absolute and relative amount of BPFD on morphology and interactions between PPFD and BPFD on photosynthesis. Further studies could also investigate whether the necessary BPFD could be reduced with an increased effect on cryptochrome with a broader blue spectrum or the addition of other wavelengths.

The alternative scenarios showed that the amount of necessary BPFD of the emitted light could be reduced through increased reflection of the bottom and soil and by changing the amount of BPFD during the growth period. Within the used LED modules, the blue LEDs had a higher energy consumption than the red LEDs, as expected from theory [6]. Simulations with BPFD levels optimized for the alternative chamber design showed the potential to decrease energy consumption. Additionally, decreased BPFD can increase water use efficiency by decreasing stomatal conductance [5]. The simulations showed a high potential for light optimization in indoor crop production and speed breeding as the model can be adjusted to the dimensions, LED types and placements and reflective properties for a system-specific recommendation for the light spectrum. Further development of the model could include response functions to more wavelengths and light intensities and make the model sink-source driven [33].

## 4. Materials and Methods

### 4.1. Experimental Setup

Soybean plants were grown inside three LED chambers (Compled Solutions GmbH, Dresden, Germany) with the dimensions: 1.1 m high, 0.5 m wide and 0.7 m deep inside a larger climate chamber at the University of Hohenheim (Germany). The LED chambers had openings at the top and bottom enabling ventilation to keep a constant temperature around 27 °C. Seeds of the soybean (*Glycine max* (L.) Merr.) cultivar Merlin (Saatbau Linz eG, Leonding, Austria) were inoculated (Soya BeanInoculant, Legume Technology Ltd., Nottinghamshire, UK) and sown in a mixture of peat substrate (Substrat 5 + Perlite; Klasmann-Deilmann GmbH, Geeste, Germany). The initial three plants per pot were thinned to one plant according to homogenous development on day nine by the start of the plant measurements. Twelve pots (9.5 × 9.0 × 9.0 cm) were distributed evenly within each chamber (0.35 m^2^) resulting in a plant density of around 34 plants m^−2^. The twelve pots were placed in a common tray and irrigated regularly to avoid water limitations. The experiment consisted of four runs within three LED growth chambers to achieve two repetitions for each of the six light treatments.

### 4.2. Light Treatments

**Figure 6:**
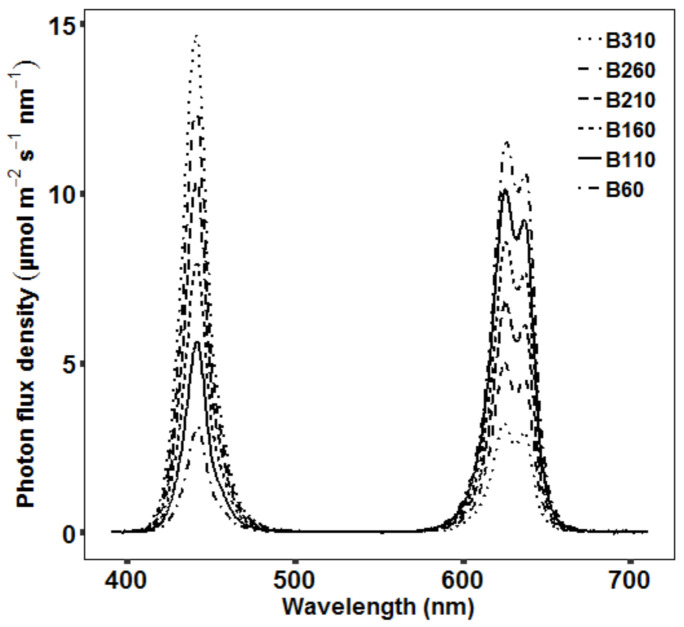
The measured spectrum of the six treatments with a BPFD of 60, 110, 160, 210, 260 and 310 µmol m^−2^ s^−1^.

Within each chamber, four LED modules (Sunsim VIS_v3; Compled Solutions GmbH, Dresden, Germany) were placed which allowed to adjust light intensity for the different wavelength ranges. The applied light treatments were comprised only of red and blue light, with one peak in the blue range at 440 nm and two peaks in the red range at 620 and 640 nm (Figure 6). The day length was set to 10 h and all treatments had a PPFD of 400 µmol m^−2^ s^−1^ to test the influence of different levels of BPFD independent from changes in PPFD. The spectrum of the six light treatments had a BPFD of 60, 110, 160, 210, 260 and 310 µmol m^−2^ s^−1^ (B60–B310) (Figure 6), while the remaining PPFD was delivered by the red LEDs. The light spectrum did not include any far-red light to exclude differences in phytochrome-mediated responses between the light treatments. Setting of the spectral intensity of the treatments was performed at 80 cm distance from the LED modules according to the measurements from a FLAME-S-XR1-ES spectrometer (Ocean Optics Germany GmbH, Ostfildern, Germany). The spectrometer measured in the range from 200–1025 nm with a resolution of around 2 nm and was equipped with a collimating lens (74-UV-MP) and a right-angle reflector with cosine corrector (74-90-UV-CC3). The photon flux density was recorded for 400–700 nm (PPFD), 400–500 nm (BPFD) and 600–700 nm (red light).

Energy consumption of the treatments was measured with a volt-ohm meter (Voltcraft, Energy Check 3000, Conrad Electronic SE, Wernberg-Köblitz, Germany). The measurements were reasonable as compared to the estimated energy consumption given by the software of the LED chambers.

### 4.3. Plant Measurements

Biomass and leaf area were measured on five dates during each run. For the first four measurements (on day 9, 13, 16 and 20), two plants were randomly selected, and the remaining four plants were used for the final measurement on day 23. On each of the five dates, total plant height (from soil to apical bud), and leaf area and biomass of each phytomer of the two/four plants were determined. Leaf area was estimated with ImageJ [50] from pictures of the leaves and dry mass was measured separately for internodes, petioles and leaf laminas after drying for at least 48 h at 60 °C until constant weight.

On day 23—when start of flowering was observed under all treatments—additional measurements of the photosynthetic rate and SPAD values were performed on the remaining four plants. The SPAD, which is representative for chlorophyll content, was measured using a SPAD meter (SPAD 502 Plus, Konica Minolta, Inc., Tokyo, Japan) and the photosynthetic rate was measured on the youngest fully developed leaf on each of the four plants per light treatment with a LCpro-SD portable system (ADC BioScientific Ltd., Hoddesdon, UK). Measurements were performed under ambient conditions within the chambers (clear glass cover to measure under the applied light treatments). Values were recorded when a steady photosynthetic rate was reached (after around 20 min).

LMR was calculated from leaf biomass/above ground biomass and IMRS was calculated from internode biomass/biomass of stalk (internode plus petiole).

Morphological measurements were performed at seven dates (day 9, 11, 13, 16, 18, 20, and 23) on the four plants within each chamber used for the final measurements. These measurements were used for calibration of the FSP model of soybean and for statistical analysis of the influence of BPFD on growth dynamics. They comprised length and diameter of internodes and petioles, length and width of leaflets, angle between internodes and petioles and angles of the leaf lamina. The latter comprising the lamina inclination measured from the base to the tip of the lamina, and the rotation angle around the midrib. To describe the unfolding of the leaf lamina, the angle between the midrib and each of the two halves of the leaf lamina was determined. Diameters were measured with a caliper, length and width with a ruler and angles with a protractor.

### 4.4. Statistical Design and Analysis

Measurements of growth dynamics of internodes, petioles and leaflets of each phytomer were used to fit the beta-function (Equation (3)) [51]:

(3) $$L\left(t\right)=L_{max}\left(1+\frac{t_{e}-t}{t_{e}-t_{m}}\right){\left(\frac{t}{t_{e}}\right)}^{\frac{t_{e}}{t_{e}-t_{m}}}\;\;0\leq t_{m}<t_{e}\;\;L\left(t>t_{e\;}\right)\;=\;L_{max}$$

where *L(t)* is the size at day *t*, *L_max_* is the final size, *t_e_* is the day when the final size is reached and *t_m_* is the day on which the growth rate peaks. The parameters *L_max_*, *t_e_* and *t_m_* were estimated with the nls-function in the R-package stats [52].

**Figure 7:**
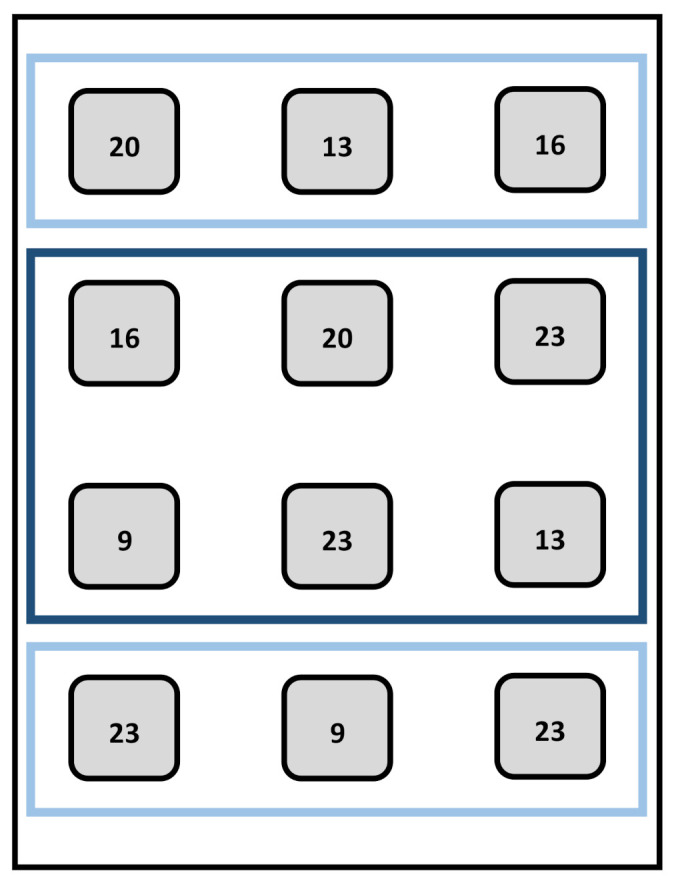
Illustration of the randomization for the plants used for the destructive measurements within the first (light blue square) and second (dark blue square) block (plant location). The numbers exemplarily show the day of the destructive measurements the plants were used for.

To determine the plants used for the destructive measurements during each experiment, randomizations were performed within two blocks (plant location). The first block comprised the first and last row and the second block the two center rows (Figure 7).

The six light treatments of the experiment were performed with two replicates. Given three LED chambers, three out of the six light treatments could be tested in the same run, i.e., each replicate comprised two runs, resulting in four runs in total (Supplementary Materials, S1). For the arrangement of treatments within the LED chambers over time and space, an α-design with two replicates and a block (time) size of two was used. The effect of BPFD on biomass, morphology, leaf physiology and parameters of the beta-function was tested. The second and mostly third phytomer (hypocotyl counted as first phytomer) were chosen for specific analysis, because they comprised the most comprehensive measurements from beginning to end of growth. According to the experimental design, the following mixed model was used to analyze the data in the SAS^®^ software (SAS Institute, Inc., Cary, NC, USA):

(4) $$y_{iklmn}=\mu +b_{k}+i_{kl}+p_{klm}+r_{klmn}+\tau_{i}+e_{iklmn}$$

where *µ* is the intercept, *b_k_* is the fixed effect of the *k*^th^ complete replicate, *i_kl_* is the random effect of the *l*^th^ incomplete block (time) within the *k*^th^ replicate, *p_klm_* is the random effect of the *m*^th^ chamber within the *l*^th^ run, *r_klmn_* is the random effect of the n^th^ block (plant location) within the *m*^th^ chamber of the *l*^th^ run and the *k*^th^ replicate, *τ_i_* is the main effect of the *i*^th^ light treatment, and *e_iklmn_* is the error effect of observation *y_iklmn_* with homogeneous variance. Residuals were checked graphically for normal distribution and homogeneous variance. After finding significant effects via F-test, a multiple t-test to compare least square means was used to create a letter display [53]. Note that least square means are presented in the results section as data was not balanced, because only three out of six light treatments were tested within each run. Least square means are based on model (Equation (4)) to adjust for block effects.

### 4.5. FSP Model

An existing 3D model of the LED growth chamber [54] in the modelling platform GroIMP [55] was used. The virtual LED chamber can be adjusted in its dimensions and the placement of the LED modules and proved to simulate the spectral light distribution with a high accuracy [54]. The single LED types are defined by their spectral and physical light distribution and total emitted power. Then, individual LEDs can be placed according to their position within the LED module. The simulations of the spectral light distribution were performed with the integrated spectral Monte-Carlo ray tracer GPUFlux [56] set to a spectral resolution of 5 nm within the 400–800 nm range. The optical properties (reflection, absorption and transmittance) of the sidewalls were zero transmission and an absorption of 0.02 within the 400–600 and 700–800 range and 0.04 within the 600–700 range.

The optical properties of the chamber were not changed from the setting in the original model [54] as the side wall material was the same. The optical properties of the soybean leaf and the substrate were set in a 5 nm resolution according to measurements from a typical soybean leaf ([57], Supplementary Materials, Figure S3) and peat [58]. The adjustments of the original model of the virtual LED chamber were location and intensity of the individual LEDs and the location of the LED modules. The intensity of the virtual LEDs were parameterized to emit the same intensity of red and blue light at 80 cm distance as in the experimental treatments (Supplementary Materials, Figure S4) using virtual sensors [54]. The virtual LEDs were set to emit 25 million rays with a maximum of 50 reflections, ensuring that all rays were absorbed by an object or reflected outside of the virtual chamber before reaching the maximum number of reflections.

Within the virtual LED chamber, an FSP model of soybean was constructed based on the generic model FSPM-P [27]. Internodes and petioles of the virtual plants were constructed as simple cylinder objects, while the shape of the leaf lamina was triangulated, based on a picture of a soybean leaflet and was composed of 42 triangles (Supplementary Materials, Figure S5).

Each leaflet was constructed from two half leaflets enabling unfolding of the leaflet from the midrib according to measurements (Supplementary Materials, Figure S5A,B). Simultaneously with the unfolding of the two leaflet halves, the leaflet moved from a vertical position with the leaflet tip pointing upwards towards a final inclination according to the measurements from leaflet base to tip and rotation around the midrib according to measurements from one side of the leaflet to the other (Supplementary Materials, Table S2; Figure S5C). The leaflet unfolded with 0.7°/hour.

The virtual plants grew according to the found growth parameters of the beta-function to simulate the plant structure according to the experimental observations. Additional inputs taken from the experiment were final organ length, petiole angles and length to diameter ratio of internodes for each treatment and ratio between length of side leaflets and center leaflet of the trifoliate leaf, leaflet length to leaflet area ratio and length to diameter ratio of petioles as an average of all treatments (Supplementary Materials, Table S2).

According to the experimental design, twelve virtual plants were simulated at the beginning, and then during the simulation two of them were randomly chosen within the two blocks to be taken out of the virtual scene on day 9, 13, 16, and 20, respectively.

### 4.6. Response Function

The response function was derived and integrated following four steps: (1) fit beta-function for all treatments, (2) run FSP model to obtain perceived BPFD of internodes for each treatment, (3) derive response function across all treatments for internode length in dependence of simulated perceived BPFD and (4) integrate the derived response function into the model for all treatments.

Based on the experimental observations, the parameters for the beta-function were derived for each light treatment. Based on these parameters, the FSP model of soybean dynamically simulated the plant architecture over time under each of the six light treatments.

At this stage, the growth of the internodes stopped at a final length according to the measurements. During the simulations, BPFD perceived by the internodes was recorded for each hour. The simulated perceived BPFD was used to fit a response function to the internode length observed under the different BPFD treatments:

(5) $$Internode\;length=L_{min}\left(1+\left(BPFD_{min}-BPFD_{per}\right)a\right)\;\;BPFD_{min}-BPFD_{per}>0$$

where *L_min_* is the minimum possible length of the internode, *BPFD_min_* is the lowest BPFD giving *L_min_*, *BPFD_per_* is the average perceived BPFD of the internode during the first four days of growth and *a* is the slope of the response to *BPFD_per_*. The first four days of growth were used due to a very rapid growth inhibiting effect of blue light [59] and according to the results of Kahlen and Stützel [46] who found four successive days starting one week before reaching maximum growth rate to be particularly sensitive to changes in PPFD.

The found BPFD response function was integrated in the FSP model to determine *L_max_* of the beta-function and the final internode length. They were hereby simulated in dependence of the perceived BPFD during the simulations and at this stage no longer determined by experimental measurements. Internode two until nine elongated according to the integrated response function, with *L_min_*, and *t_m_* set for each internode according to the found parameter values for the treatment B310 (baseline scenario with shortest internodes). Because the first internode (hypocotyl) grew before thinning and beginning of experimental measurements, its length was set to grow to the final average length of all treatments.

### 4.7. Model Evaluation and Alternative Scenarios

The light simulations were evaluated in an earlier study by comparing light measurements and simulations within a soybean canopy grown under two treatments identical to this study (B110 and B160) [38]. The integration of the parameters of the beta-function used to calibrate the dynamic model in this study was evaluated by comparing length over time for internodes, petioles and leaf laminas at all phytomer levels under all treatments. Model simulations after integration of the response function to blue light were evaluated by comparing the measured and simulated plant height until the third phytomer (hypocotyl counted as first phytomer). The comparison included the eight plants per chamber selected for the first four dates of destructive measurements, which were not used for the model parameterization.

Then, the model was applied for spectral optimization with the aim of minimizing BPFD emitted by the LED modules to reduce energy consumption, but still reach the minimum internode length. The first alternative scenario was run with a different virtual LED chamber design for evaluating the effect on the perceived BPFD. The chamber design was changed by setting the reflection of the bottom, pots and substrate to the same level as the sidewalls of the chamber. This was chosen for simulating a situation similar to e.g., hydroponics with plants placed in more reflective containers than the substrate and black pots in the experiment. This change in chamber design was expected to increase the perceived BPFD and hereby reduce BPFD emitted by the LEDs that is necessary to induce short plants. Simulations from the first scenario indicated that the spectrum could be optimized according to the developmental stage. The suggested optimization was applied in the second scenario by changing the emitted BPFD during the growth period.

## 5. Conclusions

The length of internodes and petioles increased under low BPFD, similar to the shade response under low PPFD, whereas the limited response of SLA and internode diameter indicated that the shade responses of these might not be regulated by cryptochrome. Further studies could investigate alternative regulation of these together with extended photosynthetic measurements over time to increase the understanding of carbon assimilation and translocation under different BPFD levels. Several aspects of the exact spectral effects on morphology and physiology should be further investigated, both for narrow peaks independent and the interactions with broader spectra.

Internode length dependent on perceived BPFD was well simulated in the FSP model and the simulations gave an increased insight into the response of the second and third internode based on the perceived BPFD. The model was a useful tool to determine the minimum necessary BPFD within an alternative chamber environment. Modelling with an FSP can be applied for further optimizations of indoor plant production implementing advances in knowledge of spectral effects on plant morphology and physiology.

## Figures and Tables

**Table 1:** Least square means of the final measurement of organ biomass, leaf mass ratio (LMR) and internode mass ratio of the stalk (IMRS) at phytomer scale under different blue photosynthetic flux densities (BPFD). Letters indicate significant differences between treatments (*p* < 0.05).

Phytomer Level	Organ/Ratio	Treatment					
		B60	B110	B160	B210	B260	B310
Second	Internode (g)	0.082 ^a^	0.082 ^a^	0.076 ^a,b^	0.058 ^b,c^	0.047 ^c^	0.049 ^c^
Third	Internode (g)	0.041 ^a^	0.044 ^a^	0.046 ^a^	0.030 ^b^	0.022 ^b^	0.023 ^b^
	Petiole (g)	0.016 ^a^	0.018 ^a^	0.018 ^a^	0.015 ^a^	0.013 ^a^	0.013 ^a^
	Leaf lamina (g)	0.127 ^a^	0.136 ^a,b^	0.116 ^a,c^	0.100 ^a,c^	0.078 ^c^	0.081 ^b,c^
	LMR	0.69 ^a,b^	0.68 ^b,c^	0.64 ^c^	0.67 ^b,c^	0.71 ^a^	0.71 ^a^
	IMRS	0.72 ^a^	0.72 ^a^	0.71 ^a^	0.68 ^a,b^	0.62 ^b^	0.63 ^b^

**Table 2:** Least square means of the final measurement of specific leaf area (SLA), carbon assimilation (*A*) and SPAD at phytomer scale under different blue photosynthetic flux densities (BPFD). Letters indicate significant differences between treatments (*p* < 0.05).

Phytomer Level	Measurement	Treatment					
		B60	B110	B160	B210	B260	B310
Third	SLA (cm^2^ g^−1^)	324.35 ^a,b^	303.04 ^b^	327.68 ^a,b^	327.02 ^a,b^	341.41 ^a,b^	346.38 ^a^
	SPAD value	26.51 ^a^	30.08 ^a^	31.65 ^a^	28.81 ^a^	30.37 ^a^	30.92 ^a^
Youngest fully developed	*A* (µmol CO_2_ m^−2^ s^−1^)	27.49 ^a^	28.81 ^a^	27.78 ^a^	27.03 ^a^	26.79 ^a^	26.53 ^a^

**Table 3:** Least square means of the final measurement for lengths of internodes, petioles and leaves and diameter of internodes at phytomer scale under different blue photosynthetic flux densities (BPFD). Lower case letters indicate significant differences between treatments (*p* < 0.05).

Phytomer Level	Organ	Treatment					
		B60	B110	B160	B210	B260	B310
Second	Internode (cm)	6.86 ^a^	5.86 ^b^	5.39 ^b^	4.70 ^c^	4.26 ^c^	4.33 ^c^
Third	Internode (cm)	3.81 ^a^	3.41 ^a,b^	3.28 ^a,b^	2.75 ^b,c^	2.28 ^c^	2.14 ^c^
	Petiole (cm)	5.11 ^a,b^	5.08 ^a,b^	5.61 ^a^	4.80 ^b,c^	4.72 ^b,c^	4.46 ^c^
	Leaf lamina (cm)	5.25 ^a^	5.07 ^a^	5.27 ^a^	4.58 ^a^	4.49 ^a^	4.48 ^a^
	Internode diameter (mm)	3.09 ^a^	3.29 ^a^	3.52 ^a^	3.41 ^a^	3.46 ^a^	3.28 ^a^

**Table 4:** Least square means of estimated parameters of the beta-function. Time of elongation (te) and time of maximum elongation (tm) for internode, petiole and leaf at the third phytomer under different blue photosynthetic flux densities (BPFD). Letters indicate significant differences between treatments (*p* < 0.05).

Organ of Third Phytomer	Parameter	Treatment					
		B60	B110	B160	B210	B260	B310
Internode	te	13.96 ^b^	14.39 ^a,b^	14.21 ^b^	14.41 ^a,b^	14.67 ^a^	14.36 ^a,b^
	tm	5.23 ^a^	6.86 ^a^	6.60 ^a^	6.33 ^a^	6.60 ^a^	6.13 ^a^
Petiole	te	15.76 ^c^	16.03 ^b,c^	15.85 ^c^	16.47 ^a,b^	16.39 ^a,c^	16.68 ^a^
	tm	9.79 ^b^	10.25 ^a,b^	10.18 ^a,b^	10.25 ^a,b^	10.74 ^a^	10.70 ^a^
Leaf lamina	te	13.94 ^a^	14.12 ^a^	14.04 ^a^	14.11 ^a^	14.19 ^a^	13.89 ^a^
	tm	5.19 ^a^	5.04 ^a^	4.57 ^a^	4.55 ^a^	5.50 ^a^	4.95 ^a^

**Table 5:** Measured energy consumption (Watt) of the LED chambers under different blue photosynthetic flux densities (BPFD).

Treatment	Energy Consumption (W)
B60	94.4
B110	95.1
B160	96.4
B210	97.6
B260	101.7
B310	107.2

**Table 6:** Average energy consumption during 23 days of growth for the three simulated scenarios to reach the minimum plant height.

Scenario	Light Spectra	Average Energy Consumption (W)
Experimental	B310	107.2
First scenario	B260	101.7
Second scenario	B210/B260	100.1

## Supplementary Materials

The following are available online at https://www.mdpi.com/2223-7747/9/12/1757/s1, Figure S1: Plant height (A), biomass (B) and leaf area (C) per plant and under different blue photosynthetic flux densities (BPFD). Error bars indicate standard error of the mean (day 9–20: *n* = 4, day 23: *n* = 8), Figure S2: Simulated (line) and measured (points) length of petioles, internodes and leaf laminas under the treatments B310 and B60, Figure S3: The absorption, reflection and transmission of radiation (%, relative to the incident radiation) from 400–700 nm by a soybean leaf used for the optical properties of the simulated soybean leaves. Data taken from Kasperbauer (1987), Figure S4: The simulated spectra (total PFFD of 400 µmol m^−2^ s^−1^) of the six treatments with a simulated BPFD of 60, 110, 160, 210, 260 and 310 µmol m^−2^ s^−1^, Figure S5: Visualizations of the simulated unfolding (A, B) and fully developed (C) trifoliate leaf, Table S1: The spread of the six treatments within three chambers over time, Table S2: Model inputs to determine ratios and angles of organs.
