# Supplementary material for: Modelling of Soybean (Glycine max (L.) Merr.) Response to Blue Light Intensity in Controlled Environments
Source: Plants (Basel). 2020 Dec 11;9(12):1757. doi: 10.3390/plants9121757 (PMC7764200; doi:10.3390/plants9121757)
Supplement: Supplementary file 1 [file plants-09-01757-s001.pdf]

plants

MDPI

Supplementary Materials

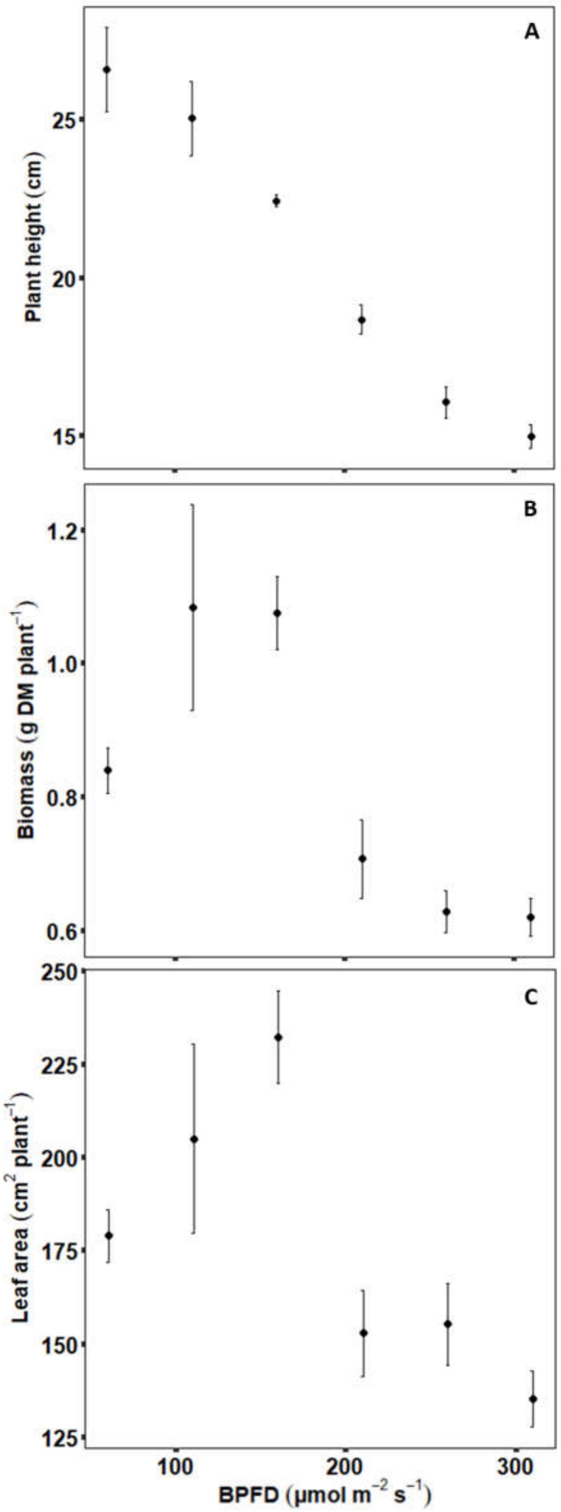

Figure S1. Plant height (**A**), biomass (**B**) and leaf area (**C**) per plant and under different blue photosynthetic flux densities (BPFD). Error bars indicate standard error of the mean (day 9-20: n=4, day 23: n = 8).

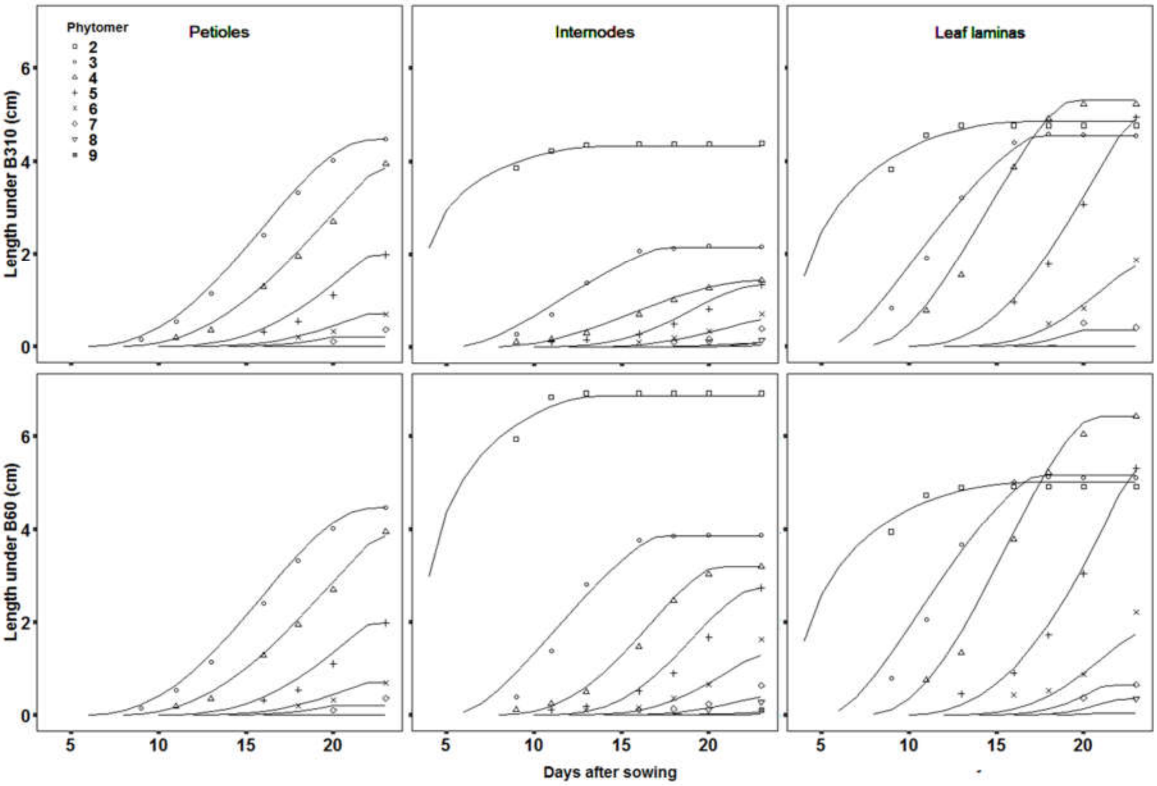

Figure S2. Simulated (line) and measured (points) length of petioles, internodes and leaf laminas under the treatments B310 and B60.

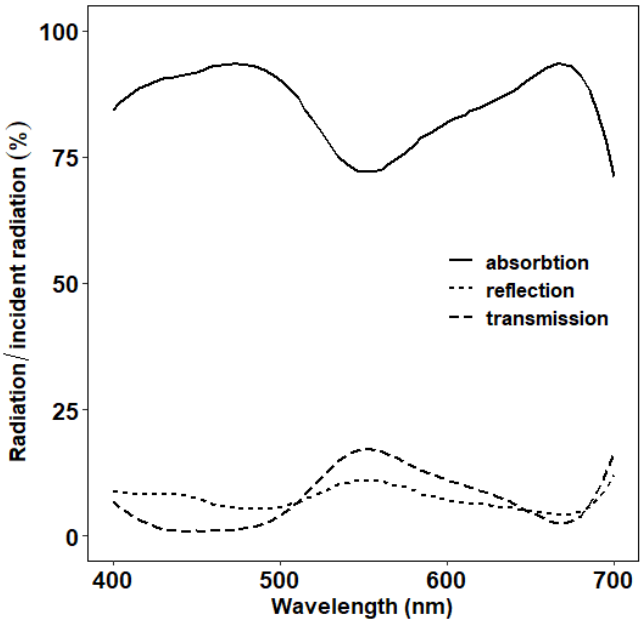

Figure S3. The absorption, reflection and transmission of radiation (%, relative to the incident radiation) from 400 – 700 nm by a soybean leaf used for the optical properties of the simulated soybean leaf. Data taken from Kasperbauer (1987).

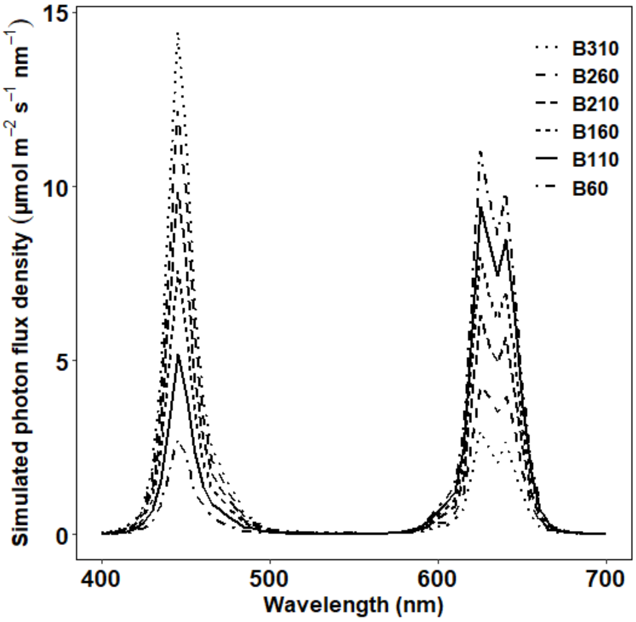

Figure S4. The simulated spectra (total PFFD of 400 μmol m^{-2} s^{-1}) of the six treatments with a simulated BPFD of 60, 110, 160, 210, 260 and 310 μmol m^{-2} s^{-1}.

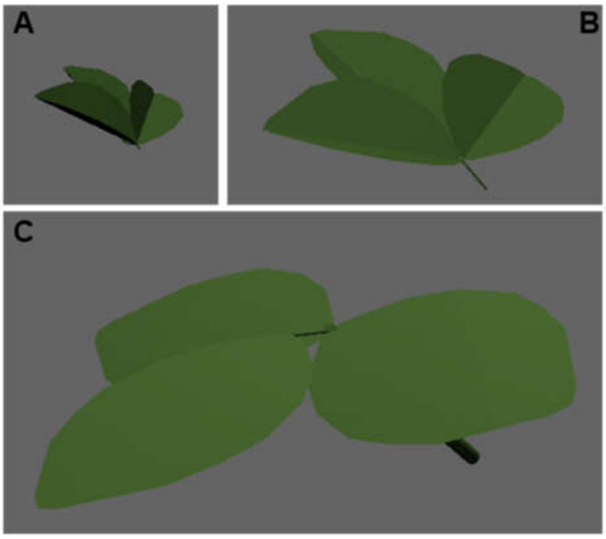

Figure S5. Visualizations of the simulated unfolding (**A**, **B**) and fully developed (**C**) trifoliate leaf.

Table S1. The spread of the six treatments within three chambers over time.

Repetition	Run	Chamber	Treatment
1	1	1	B260
		2	B60
		3	B310
	2	1	B160
		2	B210
		3	B110
2	3	1	B210
		2	B60
		3	B110
	4	1	B260
		2	B310
		3	B160

Table S2. Model inputs to determine ratios and angles of organs.

Plant Level	Parameter	Treatment					
		B60	B110	B160	B210	B260	B310
All phytomers	Side leaflet : center leaflet length	0.94	0.94	0.94	0.94	0.94	0.94
	Internode diameter : length	0.034	0.049	0.058	0.066	0.079	0.077
	Petiole diameter : length	0.03	0.03	0.03	0.03	0.03	0.03
	Petiole angle	26	23	33	32	38	34
Second phytomer	Leaf inclination angle	45	46	42	34	58	46
	Leaf rotation angle	1.3	3.8	0.6	5.6	1.9	11.3
Third phytomer and higher	Center leaflet inclination angle	44	36	34	24	42	23
	Center leaflet rotation angle	19	12	14	9	18	4
	Side leaflet inclination angle	34	33	27	18	29	9
	Side leaflet rotation angle	41	11	16	24	39	33
